# Notes on Therapeutics

**Published:** 1883-01

**Authors:** R. L. MacDonnell

**Affiliations:** Assistant Demonstrator of Anatomy, McGill University, Montreal


NOTES ON THERAPEUTICS. By R. L. MACDONNELL, B.A., M.D., M.R.C.S., Assistant Demonstrator of Anatomy, McGill University, Montreal.

Every medical man has his share of the worry and annoyance arising from the treatment of hysterical and allied disorders. He who has an hysterical spine in his practice cannot be happy. He is doomed to be supplanted by the hydropathist, the homœopathist, or the movement cure man. In Montreal, we are constantly hearing of such cases being cured by faith, and, inasmuch as the miracle is much advertised in the evening papers, we are assured of a fine supply of such cases in the future. What bliss it must be for the hystero-malingero hypochondriac to read the record of her sufferings in a newspaper, her early history, her devotion, her domestic virtues, how many doctors came, saw, and shook their heads. The Weir Mitchell plan of treatment has come to the rescue, though, and will undoubtedly do much to keep the neurasthenic out of the hands of the quack.

The profession in England seem to hold a very high opinion of this line of practice. The "Systematic Treatment of Aggravated Hysteria and Certain Allied Forms of Neurasthenic Disease," was the title of a paper read by Dr. Playfair * to open the discussion upon this subject at the meeting of the British Medical Association at Worcester last August.

Dr. Playfair has had eighteen months' experience of this treatment, and has not only acquired a daily increasing confidence in the value of Weir Mitchell's method, but has had more satisfactory and surprising results from it than he has ever before witnessed in any branch of his professional experience, and now he more confidently undertakes the care of a well selected case of this kind, than he does that of almost any other malady that comes under his notice.

When he talks of the "useless system of drugging with so called nerve tonics," one feels inclined to cry "hear, hear." At the same time that we are giving these medicines, the psychological causes are all at work, "the injudicious and constant nursing, the craving for sympathy, the fact that the sick-room becomes the center of interest for the patient and her friends, the constant discussion of feelings and symptoms." At this period of pessaries, of lacerations, of flexions, which has succeeded the "ulcerated os" period, and now, when every pain, every ache, every passing abnormal sensation, is pounced upon by the gynæcologist as appertaining to his special field of work, it is highly comforting to read the words of one so eminent in that branch of medicine as Dr. Playfair. "It would be a great mistake, however, to conclude that there is any necessary or constant connection between the two. Indeed, although very frequently the nerve state has originated in connection with uterine disease, in a large proportion of the cases I have seen, it has completely overshadowed the original local disorder. I am not sure that I should not, in common honesty, make the somewhat humiliating confession, that, in many instances, overmuch and injudicious local treatment has, in my opinion at least, intensified and kept up the now dominating neurasthenic disorder, as in a

* *British Medical Journal*, August 19, 1882.

case under my care as I write, in which the patient may fairly be said to be suffering from pessary on the brain—so incessantly is she thinking of one or other of the seventy-nine different instruments she has had inserted in the last few years in America and in this country."

The case best suited for systematic Weir Mitchell treatment is the worn and wasted, often bedridden woman, who has broken down, either from some sudden shock, such as grief, or money losses, or excessive mental or bodily strain, beginning with simple debility more and more yielded to, until at last all power of effort is lost. Coincident with this is the total loss of appetite, the profound anæmia, and the consequent wasting of the tissues. Then follow the graver forms of hysterical disease, such as paresis, or paralysis, vomiting, disorder of motion, hystero-epilepsy, and many others which constitute the despair of the physician.

The principal elements in the systematic management of these cases are:

1. The removal of the patient from unhealthy home influences, and the placing her at absolute rest. 2. The production of muscular waste, and the consequent possibility of assimilating food, by what have been called "mechanical tonics," viz.: prolonged movement and massage of the muscles by a trained shampooer, and muscular contractions produced by electricity. 3. Supplying the waste so produced by regular and excessive feeding, so that the whole system, and the nervous system in particnlar, shall be nourished in spite of the patient.

Dr. Playfair cites four cases to illustrate the class of disease to which this method is applicable, and the uselessness of all ordinary treatment in such conditions. In one of his cases no less than twenty-five medical men had been consulted, this number including the names of many of the most eminent consultants in the country, of itself a sufficient proof that all that the most advanced medical knowledge and skill could do had been tried in vain.

Case 1. A young lady with hysterical vomiting, which she had had for six years. Latterly, she could keep nothing upon her stomach but a single mouthful of milk, and this only when mixed with whisky; so that in this way she was taking three to four glasses of spirit daily. Weight 63 lbs. In three days after isolation, she was keeping down two quarts of milk, without the aid of whisky. In ten days she was eating with an enormous appetite, and in six weeks she left town weighing 106 lbs., a gain of 43 lbs., and has since remained quite well.

Case II. A girl aged seventeen. Hysterical hemiplegia of four years' standing, during which time she had never been out of bed. Severe cough, which had resisted all medication. No food could be taken beyond milk, a biscuit and an orange. At the end of a month Dr. Playfair drove her out in his carriage, dropped her at the top of the street in which she lived, and made her walk down to pay her parents a visit. She has since remained quite well. The cough ceased forty-eight hours after she was removed, and was never heard again.

Case III. This next instance is an example of the kind of case best suited for this treatment. No definite illness; no simulated disease, but a general breakdown. An invalid all her life; sometimes headache and nausea; at others, spinal irritability, giddiness, etc. Never happy unless seeing a doctor or taking physic. She was wasted to a skeleton. Her chief complaints were nausea, headache, backache, intense nervous depression and timidity (so that she was unable to speak to a stranger), and absolute anorexia; skin dry and rough; menstruation irregular; entirely dependent on chloral and morphia for sleep. Had been nine years upon her back. In six weeks she was walking about. She now plays tennis, goes out to picnics and parties, and enjoys life like any one else.

Case IV. This case must be well known to many members of the profession, since there is scarcely a consultant of eminence who has not seen her during the sixteen years her illness has lasted. Dr. Playfair's acquaintance with the case is curious. He first saw on the esplanade, at Brighton, a remarkable party at which everybody was looking. The chief personage in it was a lady, reclining at full length upon a long couch, and being dragged along, looking the picture of misery, emaciated to the last degree, her head drawn back in a state of opisthotonos, her hands and arms clenched and contracted, her eyes fixed and staring at the sky. There was something in the whole procession that struck him as being typical of hysteria. She had been dragged about Brighton in this way for ten or twelve years. Dr. Playfair took charge of the case on January 14, 1882. She had exhibited the following symptoms since 1864, when she was first attacked with paralysis of the left arm: complete paraplegia; left hemiplegia; complete hysterical amaurosis. She had been bedridden since the illness began, and had not passed urine spontaneously for sixteen years. There was "awful suffering in the spine, head and eyes," requiring the use of chloral and morphia in large doses. The following are the brief notes from Dr. Playfair's note-book on the day of his first visit: "I found the patient lying on an invalid couch, her left arm paralyzed and rigidly contracted, strapped to her body to keep it in position. She was groaning loudly at intervals of a few seconds, from severe pain in her back. When I attempted to shake her right hand, she begged me not to touch her, as it would throw her into a convulsion." Said to have had epilepsy as a child. Daily and hourly attacks of loss of consciousness. The left arm and both legs are paralyzed. Takes hardly any food, and is terribly emaciated.

The first night she was in the Home Hospital, in Fitzroy Square, she shrieked and groaned so much that no one in the house was able to sleep. On the next afternoon, between 3 P. M. and 11:30 P. M., she had had nine violent convulsive paroxysms of an epileptiform character. The next day she was quieter. On the fourth day she passed urine spontaneously, and the catheter was never again used. In two months she went a sea voyage to the Cape. She now remains in robust health, and joins with pleasure in society.

In the discussion which followed, Dr. Clifford Allbut mentioned a case he had sent to Dr. Playfair from Yorkshire. She was removed to London under chloroform, swung in a hammock. She was cured in about six or eight weeks, then went on a sea voyage, and had been perfectly well ever since.

Dr. H. Bennett (Weybridge) felt inclined to break a lance in favor of the old Hippocratic doctrine, which referred hysteria in many cases to the uterine organs, especially in young females. He had introduced to the profession a fact now generally acknowledged, viz., that uterine disease, inflammatory and otherwise, was not unfrequently found in virgins. He had, in many cases, cured them merely by getting at the disease, and removing it.

Dr. Playfair in reply, said that he had not sufficient experience of the effect of the treatment in men, although, doubtless, in suitable cases it might answer well. In chorea it certainly did good. Real organic disease is a positive contra-indication. Hitherto none of his cases have relapsed.

